# Preparation and Characterization of Chitosan Obtained from Shells of Shrimp (*Litopenaeus vannamei* Boone)

**DOI:** 10.3390/md15050141

**Published:** 2017-05-15

**Authors:** Rayane Santa Cruz Martins de Queiroz Antonino, Bianca Rosa Paschoal Lia Fook, Vítor Alexandre de Oliveira Lima, Raid Ícaro de Farias Rached, Eunice Paloma Nascimento Lima, Rodrigo José da Silva Lima, Carlos Andrés Peniche Covas, Marcus Vinícius Lia Fook

**Affiliations:** 1 Academic Unit of Materials Engineering (UAEMat), Federal University of Campina Grande (UFCG), Campina Grande 58429-900, PB, Brazil; biancafook@gmail.com (B.R.P.L.F); raidicaro@gmail.com (R.Í.d.F.R.); eunicelima@outlook.com (E.P.N.L.); 2 Academic Unit of Physics (UAF), Federal University of Campina Grande (UFCG), Campina Grande 58429-900, PB, Brazil; vitorao.lima@gmail.com (V.A.d.O.L.); rodrigo@df.ufcg.edu.br (R.J.d.S.L.); marcus.fook@pq.cnpq.br (M.V.L.F.); 3 Centro de Biomateriales—Universidad de La Habana, Ave. Universidad s/n, La Habana 10600, Cuba; cpeniche2015@yahoo.com

**Keywords:** chitosan, chitin, *Litopenaeus vannamei* Boone

## Abstract

The main source of commercial chitosan is the extensive deacetylation of its parent polymer chitin. It is present in green algae, the cell walls or fungi and in the exoskeleton of crustaceans. A novel procedure for preparing chitosan from shrimp shells was developed. The procedure involves two 10-minutes bleaching steps with ethanol after the usual demineralization and deproteinization processes. Before deacetylation, chitin was immersed in 12.5 M NaOH, cooled down and kept frozen for 24 h. The obtained chitosan was characterized using scanning electron microscopy (SEM), Fourier transform infrared spectroscopy (FTIR), UV, X-ray diffraction (XRD) and viscosimetry. Samples of white chitosan with acetylation degrees below 9% were obtained, as determined by FTIR and UV-first derivative spectroscopy. The change in the morphology of samples was followed by SEM. The ash content of chitosan samples were all below 0.063%. Chitosan was soluble in 1% acetic acid with insoluble contents of 0.62% or less. XRD patterns exhibited the characteristic peaks of chitosan centered at 10 and 20 degrees in 2θ. The molecular weight of chitosan was between 2.3 and 2.8 $\times \;10^{5}$ g/mol. It is concluded that the procedure developed in the present work allowed obtaining chitosans with physical and chemical properties suitable for pharmaceutical applications.

## 1. Introduction

**Figure 1:**
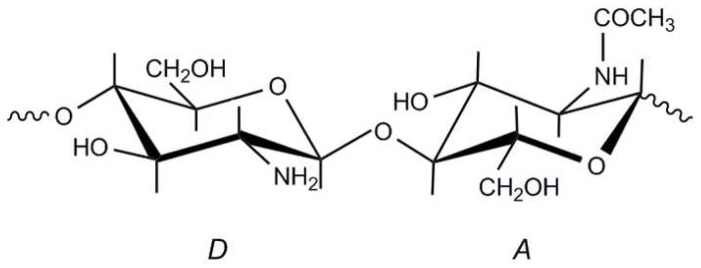
Structural units of chitin and chitosan. (*A*) *N*-acetylglucosamine unit; (*D*) glucosamine unit. In chitosan, (D)>(A); in chitin, (A)>(D).

Chitosan is a linear polysaccharide of natural origin composed essentially of β-(1,4)-linked glucosamine units (2-amino-2-deoxy-β-d-glucopyranose) together with some proportion of *N*-acetylglucosamine units (2-acetamino-2-deoxy-β-d-glucopyranose) (Figure 1). Chitosan is characterized by its biocompatibility, biodegradability and non-toxicity [1]. Its exceptional biological properties (antimicrobial, antibacterial and coagulating activities, bioadhesivity and wound healing capacity) have made it an excellent candidate for applications in cosmetics [2], medicine [1,3,4] and pharmacy [5], agriculture [6], chitosan in the preservation of agricultural commodities [7], the food industry [8] and waste water treatment [9], among many other industrial applications.

Chitosan is soluble in dilute solutions of many organic and inorganic acids (*pH < 6*), due to the protonation of its amino groups. Its polycationic character allows it to interact with polyanions producing polyelectrolyte complexes [10]. It has excellent film forming ability and can also be prepared in films, fibers, hydrogels and micro/nanoparticles [11,12]. The OH and NH${}_{2}$ functionalities in chitosan’s structure allows the preparation of diverse derivatives with improved properties for specific applications.

Chitosan is not extensively present in nature, although it can be found in in the Mucorales, in particular *Mucor*, *Absidia* and *Rhizopus* species [13]. The main source of commercial chitosan is the extensive deacetylation of its parent polymer chitin (Figure 1). Chitin is the second most abundant polysaccharide on earth, only preceded by cellulose. It is present in green algae, the cell walls or fungi, the cuticles of insects and arachnids and in the exoskeleton of crustaceans. At the industrial scale, the main source of chitin are the discarded remnants from crustacean (shrimp, prawn, crab and lobster) processing plants. The main components of crustacean shells are chitin (15–40%), protein (20–40%), calcium and magnesium carbonate (20–50%), together with other minor constituents, such as astaxanthin, lipids and other minerals [14]. The isolation techniques reported are diverse, since they depend largely on the composition of the source, which varies considerably form one species to another [15]. Most of these techniques rely on chemical processes for extracting the protein and the removal of the inorganic matter. Some of them include a bleaching step by solvent extraction or by oxidation of the remaining pigments [16].

Demineralization of crustacean shells is usually carried out with dilute HCl solutions at room temperature, although other acids have also been used (HNO${}_{3}$, H${}_{2}$SO${}_{4}$, CH${}_{3}$COOH). The acid concentration and the time of treatment depend on the source of chitin, but, in any case, high temperatures are undesirable to prevent polymer degradation [17]. Alternative treatments to prevent degradation consist in the use of ethylenediaminetetraacetic acid (EDTA) [18] or ionic liquid extraction [19,20]. Lactic acid fermentation was used to extract chitin from prawn shells, but the product obtained a low quality chitin compared with the chemical extraction method [21]. Deproteinization of shrimp shells is often performed with dilute NaOH solutions at 65–100 ${}^{\circ }$C from 0.5 to 72 h. Some procedures involve two consecutive treatments for a short time, since long time periods or high temperatures can provoke chain scission and partial deacetylation of the polymer [17]. Enzymatic extracts or isolated enzymes and microbiological fermentation have been tested with some success [22], but the alternative of the enzymatic/microbiological treatment, besides being time-consuming, also usually leaves 1–7% of residual protein [23].

Deacetylation of chitin to produce chitosan is usually achieved by hydrolysis of the acetamide groups with concentrated NaOH or KOH (40–50%) at temperatures above 100 ${}^{\circ }$C. This reaction is generally carried out under heterogeneous conditions. The acetylation degree (DA) of chitosan, defined as the proportion of acetylglucosamine units in the polymer, will depend on the deacetylation conditions. It is very difficult to completely deacetylate chitin without using specific procedures, so that the DA of chitosans generally lies between 40% and 13%, while its molecular weight ranges from $2\times 10^{5}$ to $1\times 10^{6}$ Da. The distribution of deacetylated units along the chitosan chain will also depend on the source and the preparation conditions [24]. Lertwattanaseri et al. reported that they needed to treat chitin whiskers lat reflux for 15 h in at least 40% alkaline solution to obtain chitosan with a DA below 10% [25]. In the same paper, these authors showed that, by applying the microwave technique to chitin whiskers, it was possible to achieve chitosans with DA lower than 10% in only 3 h using a 60% (*w*/*v*) alkali concentration. Biotechnological processes based on the enzymatic deacetylation of chitin have been proposed as an alternative to minimize the effects of the chemical route. Some enzymes called chitin deacetylases are used for hydrolyzing the *N*-acetamide bonds in order to obtain chitosan [26]. These enzymes can be obtained from some species of fungi and insects such as *Mucor rouxii*, *Aspergillus nidulans*, *Absidia coerulea* and *Colletotrichum lindemuthianum*. Obtaining chitosan by enzymatic reactions is an alternative for greater control of the properties and characteristics of the final product, such as degree of acetylation and average molecular mass. It is an alternative that is still at the laboratory scale, due to the sensitivity of the processing [15].

There are several reports in the literature on the preparation of chitin and chitosan from shrimp shells [27,28,29,30,31], but, as indicated above, the quality of the resulting chitosan was very dependent on the raw material and the particular procedure employed. Lamark et al. developed a multistep procedure to obtain fully deacetylated chitin preforming freeze-pump out-thaw (FPT) cycles (freezing at −193 ${}^{\circ }$C and thawing at 25 ${}^{\circ }$C) in the presence of 50% (*w*/*v*) NaOH, with deacetylation temperatures ranging from 80 to 110 ${}^{\circ }$C. The FPT cycles improved the reaction effectiveness by opening the crystalline structure of chitin, making it more permeable to alkaline solutions. This increased the accessibility of the acetylated units to the alkali and facilitated their deacetylation [32].

The shrimp species *Litopenaeus vannamei* Boone is abundant in breeding sites in northeastern Brazil, and industrial processing plants generate considerable amount of discards. This waste is a protein- and chitin-rich pollutant that can be valued if a method of extracting these shell components is designed and implemented.

In the present work, we developed a new procedure for the preparation of chitosan from shrimp (*Litopenaeus vannamei* Boone) by introducing a step in which chitin is frozen at −83 ${}^{\circ }$C in the presence of 12.5 M NaOH before deacetylation in order to increase reaction efficiency. This method was able to achieve DA lower than 10%. The chitosan obtained was characterized using scanning electron microscopy (SEM), Fourier transform infrared spectrometry (FTIR) , UV-visible spectroscopy (UV) and X-ray diffraction (XRD).

## 2. Materials and Methods

**Figure 2:**
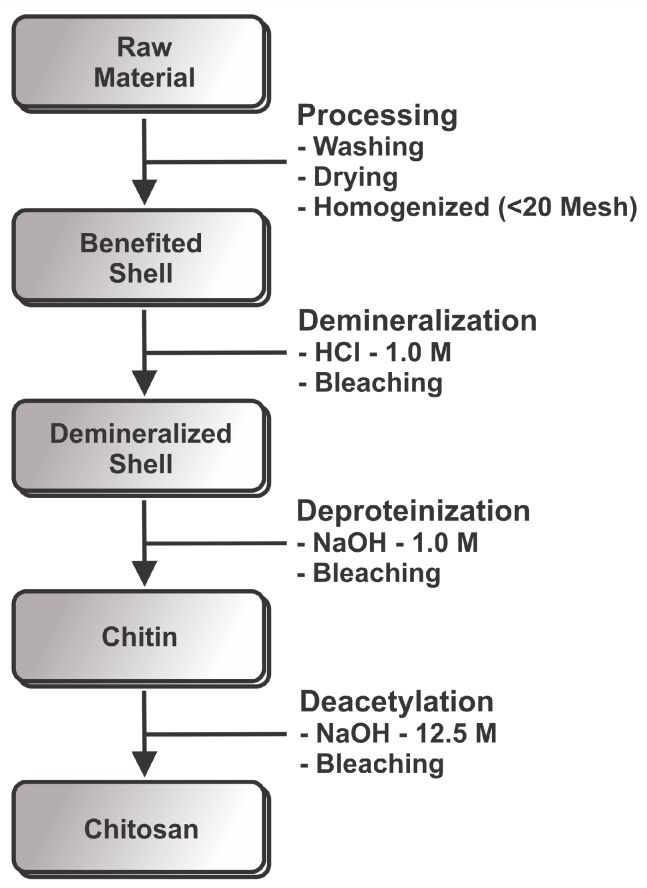
Schematic representation of the production of chitosan from shrimp.

Shells of marine Pacific whiteleg shrimp (*Litopenaeus vannamei* Boone) were obtained from Fazenda Aquamaris S.A (João Pessoa-PB, Brazil). Figure 2 shows a schematic representation of the production of chitosan from shrimp. The shells were washed thoroughly with water to remove impurities in a hot-air oven at 90 ${}^{\circ }$C for 6 h. For chitin and chitosan productions, shells were homogenized in a blender into small sized pieces (<20 mesh). This material was kept frozen until used. Acetate of sodium C${}_{2}$H${}_{3}$NaO${}_{2}$ were obtained from Grupo Química, Lot: 1093 (São Paulo, Brazil). Acetic acid glacial CH${}_{3}$COOH were obtained from Neon, Lot: 21352 (São Paulo, Brazil). Cloridric acid was obtained from Isofar, Lot: 141861 (Rio de Janeiro, Brazil). Sulfuric acid H${}_{2}$SO${}_{4}$ was obtained from Synth, Lot: 151095 (São Paulo, Brazil). Potassium bromide KBr was obtained from Neon, Lot: 3166 (São Paulo, Brazil). Ethanol C${}_{2}$H${}_{6}$O was obtained from Toscano, Lot: 259 (Santa Rita, Brazil). Sodium hydroxide NaOH were obtained from Neon, Lot: 21025 (São Paulo, Brazil). *N*-acetylglucosamine C${}_{8}$H${}_{15}$NO${}_{6}$ was obtained from Sigma Aldrich, Lot: SLBF8809V (Missouri, TX, USA).

### 2.1. Demineralization

Demineralization was carried out by adding 1 L of 1 M HCl to 100 g of shrimp shells. The reaction proceeded at room temperature under agitation at 250 rpm for predetermined times (0.5, 2, or 6 h). Afterwards, the demineralized shells were filtrated and washed with distilled water until neutral pH. They were bleached by immersing in ethanol for 10 min and dried in an oven at 70 ${}^{\circ }$C.

### 2.2. Deproteinization

Deproteinization was performed by adding 1 M NaOH to the dried demineralized shells at a solid/liquid ratio of 1:10 (g/mL). Reaction was carried out under agitation at 80 ${}^{\circ }$C for 3 h. The solid was filtrated and washed with distilled water until it achieved neutral pH. Then, it was immersed in ethanol for 10 min for further bleaching, and the resulting chitin was dried in an oven at 70 ${}^{\circ }$C.

### 2.3. Chitosan Production

Deacetylation of chitin was achieved by reacting chitin with 12.5 M NaOH at a solid/liquid ratio of 1:15 (g/mL). The reaction mixture was cooled down and kept frozen at −83 ${}^{\circ }$C in an ultra-freezer for 24 h. Afterwards, the temperature of the mixture was raised to 115 ${}^{\circ }$C, and the reaction proceeded with agitation at 250 rpm for 4 or 6 h. The resulting chitosan was filtrated, washed with distilled water until neutral pH and dried in an oven at 70 ${}^{\circ }$C.

### 2.4. Characterization of Samples

#### 2.4.1. Sulfated Ash and Insuluble Content

The amount of inorganics in samples was determined as described in the Brazilian Pharmacopeia [33]. Briefly, 1 g of sample was placed in a porcelain crucible previously ignited and weighed. The sample was moistened with 1 mL of concentrated H${}_{2}$SO${}_{4}$ and gently heated until the sample was thoroughly charred. After cooling, another 1 mL of H${}_{2}$SO${}_{4}$ was added to the crucible. The crucible was heated lightly to (600±50) ${}^{\circ }$C and maintained at this temperature until the residue became completely incinerated. The crucible was cooled in a desiccator and weighed. The operation of heating and cooling was repeated until constant weight was obtained. The content of sulfated ashes was calculated as

(1) $$SulfatedAsh\left(\%\right)=\frac{\left(W_{2}-W_{1}\right)}{W}\times 100,$$

where *W*, $W_{1}$ and $W_{2}$ are the weights of the sample taken for the test, the ignited crucible and the ignited residue with the crucible. Determinations were performed in triplicate and the results reported are the average values.

The solubility of chitosan was tested in dilute solutions of 1% acetic acid at 25 ${}^{\circ }$C. The insoluble content was determined in triplicate, as established in ASTM F2103-11 [34]. Briefly, chitosan was dissolved in 1% of acetic acid at 25 ${}^{\circ }$C and the solution was filtered. Insoluble content was calculated form the weight of chitosan dissolved and the weight of insoluble particles obtained on the filter.

#### 2.4.2. Scanning Electron Microscopy

Morphology of samples was inspected using a Hitachi scanning electron microscope model TM-1000 (Tokyo, Japan) operated at an acceleration voltage of 15 kV. The microscope is equipped with an energy dispersive spectroscopy (EDS) detector (Oxford instruments, Oxford, UK). The samples for SEM were prepared by depositing onto carbon tape.

#### 2.4.3. Viscosity Average Molecular Weight

Viscosity average molecular weight ($M_{v}$) was obtained by determining the intrinsic viscosity [η] of chitosan solutions in 0.3 M acetic acid/0.2 M sodium acetate at 25 ${}^{\circ }$C using a previously reported method [35]. Determinations were performed in triplicate using an Ubbelohde type viscometer and $M_{v}$ was calculated using the Mark–Houwink–Sakurada equation:

(2) $$\left[\eta \right]=KM_{v}^{a}.$$

In this chitosan-solvent system, K=0.076 mL/g and a=0.76.

#### 2.4.4. Degree of Acetylation

The degree of acetylation (DA) was determined in triplicate by Fourier Transform Infrared Spectroscopy (FTIR). FTIR spectra were recorded at room temperature using a 400 Perkin Elmer spectrometer (Perkin-Elmer, Norwalk, CA, USA) from 4000 cm${}^{-1}$ to 400 cm${}^{-1}$. Samples were thoroughly dried and ground with KBr at a sample/KBr ratio of 1:60. Discs were prepared by compression under vacuum. Spectra were obtained with a resolution of 2 cm${}^{-1}$ and were averaged over 100 scans. DA was calculated from the spectra using Equations (3)–(5), as proposed by Domszy and Roberts and Brugnerotto et al. [18,36], respectively:

(3) $$DA\left(\%\right)=\left(\frac{A_{1655}}{A_{3450}}\right)\times 100/1.33,$$

(4) $$DA\left(\%\right)=\left(\frac{A_{1655}}{A_{3450}}\right)\times 115,$$

(5) $$\frac{A_{1320}}{A_{1420}}=0.3822-0.03133DA\left(\%\right).$$

In these equations, $A_{1655}$, $A_{3450}$, $A_{1320}$ and $A_{1420}$ represent the absorbance of chitosan at 1655, 3450, 1320 and 1420 cm${}^{-1}$, respectively.

The DA was also determined in triplicate by the first derivative ultraviolet spectrophotometry method as described by Muzzarelli and Rochetti [37]. The principle of this method was based on the absorbance of the intensity of the *N*-acetyl group in chitosan. The method requires the determination of a standard curve produced from varying concentrations of *N*-acetylglucosamine. DA was calculated from recordings of the first derivative of the UV spectra of *N*-acetylglucosamine and of chitosan samples at 202 nm. Measurements were performed with a Cary 50 Bio Varian spectrophotometer (Palo Alto, CA, USA) with a 190–240 nm scan range. Far UV quartz cuvettes with 1 cm path length were used.

#### 2.4.5. X-ray Diffraction

Crystallinity of samples was evaluated by wide angle X-ray diffraction (WAXD) analysis using an XRD 7000 Shimadzu (Shimadzu, Kyoto, Japan) difractometer operated with Cu Kα radiation (λ=0.15418 nm). Diffraction patterns were recorded over a 2θ range of 5${}^{\circ }$–40${}^{\circ }$ in continuous mode. The step size was 0.02${}^{\circ }$. The crystallinity index (CrI) was obtained from the ratio of the area of the crystalline contribution ($A_{crist}$) to the total area of the diffractogram ($A_{total}$) as proposed by Osorio-Madrazo et al. [38]. For doing so, $A_{crist}$ was first obtained by subtracting the amorphous contribution from $A_{total}$:

(6) $$\%CrI=100\times A_{crist}/A_{Total}.$$

## 3. Results and Discussion

### 3.1. Sulfated Ash and Insoluble Content

Chitosan samples were prepared with the different reaction conditions chosen. They were labeled as shown in Table 1. All chitosan were obtained as white powders evidencing the efficiency of the bleaching treatments performed after the demineralization and deproteinization steps.

### 3.2. Scanning Electron Microscopy

**Figure 3:**
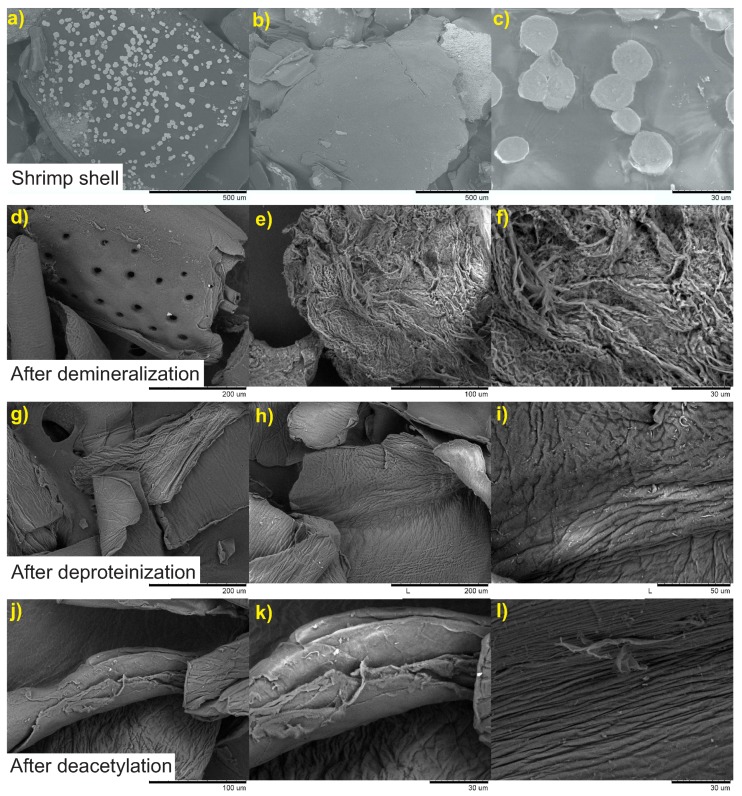
SEM micrographs of shrimp shells and the materials obtained with the processing conditions used for sample CHI-4 at three magnifications. Shrimp shell: (**a**) 200×; (**b**) 150×; (**c**) 2000×; after demineralization: (**d**) 500×; (**e**) 1000×; (**f**) 2000×; after deproteinization: (**g**) 500×; (**h**) 500×; (**i**) 1500×; after deacetylation: (**j**) 1000×; (**k**) 2000×; (**l**) 2000×.

The morphology of the starting material and the effect of each reaction step on the morphology of samples were inspected by SEM. These are shown in Figure 3.

Shrimp shells exhibit a heterogeneous morphology characterized by a compact structure with well-defined round shaped white spots (Figure 3). EDS analysis of these spots (results not shown) indicated that they are composed almost exclusively by CaCO${}_{3}$. After demineralization, one can appreciate important changes in the surface of material. The white spots are now replaced by rounded holes resulting from the removal of CaCO${}_{3}$ by the acid treatment. EDS analysis showed the absence of Ca in the demineralized shells. In higher magnification, the fibrous nature of the material is revealed. The deproteinization step of the demineralized shells produced chitin. Its fibrous structure is also revealed in Figure 3. SEM micrographs of deacetylated sample CHI-4 also show the fibrous structure of the chitosan obtained. It is worth mentioning that all of the samples of the chitosan produced by processes employed to obtain chitosan (Table 1) exhibited similar morphological behavior.

All chitosans gave clear transparent solutions in 1% (*v*/*v*) acetic acid with very low insoluble content, as reported in Table 1. The sulfated ash content was lower than 0.1% percent in all cases. These two parameters are within the requirements for chitosan applications in biomedical and tissue-engineered medical products [39,40].

### 3.3. Degree of Acetylation

**Figure 4:**
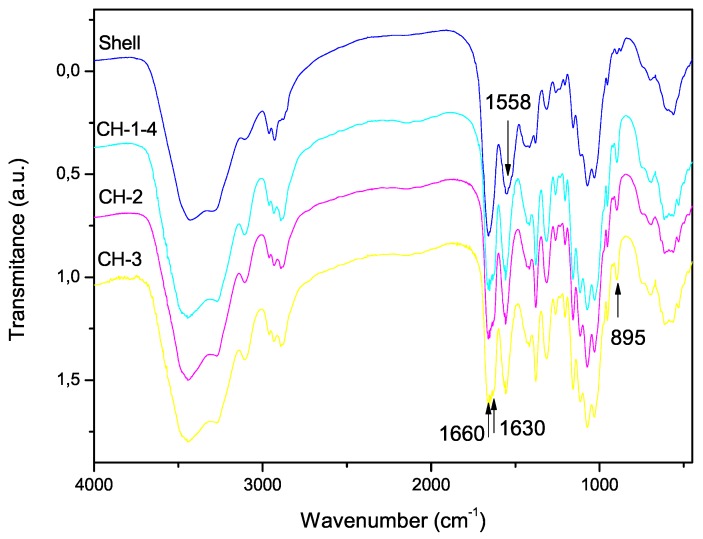
FTIR spectra of shell shrimp and chitin samples CH-1-4 with demineralization of 6 h, CH-2 with demineralization of 2 h and CH-3 with demineralization of 0.5 h.

**Figure 5:**
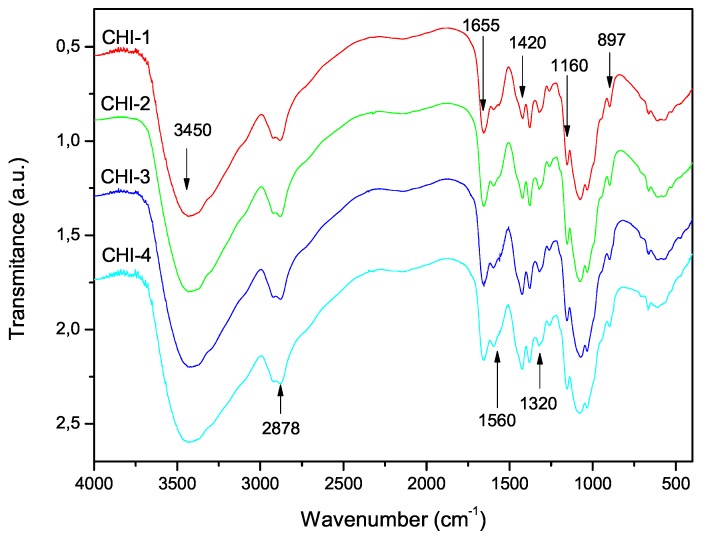
FTIR spectra of chitosan samples prepared with the reaction conditions listed in Table 1.

The FTIR spectra of the shell shrimp and chitin samples obtained are shown in Figure 4 and chitosan in Figure 5. Figure 4 shows α-chitin characteristic bands such as: amide I band is split into two components at 1660 and 1630 cm${}^{-1}$ and amide II band is observed at 1558 cm${}^{-1}$. The CH deformation of the β-glycosidic bond is centered at 895 cm${}^{-1}$ [41]. Figure 5 exhibits the characteristic absorption bands at 3450 cm${}^{-1}$ (O-H stretching), 1870–2880 cm${}^{-1}$ (CH-stretching), 1655 cm${}^{-1}$ (Amide I), 1580 cm${}^{-1}$ (–$NH_{2}$ bending), and 1320 cm${}^{-1}$ (Amide III). The absorption bands at 1160 cm${}^{-1}$ (anti-symmetric stretching of the C-O-C bridge), 1082 and 1032 cm${}^{-1}$ (skeletal vibrations involving the C-O stretching) are characteristic of its saccharide structure [41].

Equations (3)–(5) were used to determine the degree of acetylation of chitosan samples from the infrared spectra. The results are listed in Table 2, together with the acetylation degree obtained by UV-first derivative method. The degrees of acetylation were all below 9% for all samples. It is worth mentioning that the present procedure differs from other heterogeneous deacetylation procedures in that chitin samples are immersed in strong alkaline medium (12.5 M NaOH) and frozen for 24 h before starting deacetylation. This previous treatment must be responsible for the low acetylation values achieved in all cases.

It is evident that the DA values obtained were dependent on the method of calculation. They are in the order Equation (3) < Equation (4) < Equation (5) < UV-first derivative. Since the latter has been recognized as the most sensitive of the proposed techniques for determining the DA of chitosan [42], the equation proposed by Brugnerotto et al. [18] resulted in the most appropriate one for determining the DA of these samples by FTIR.

### 3.4. Viscosity Average Molecular Weight

The intrinsic viscosity, [η], of chitosan solutions in 0.3 M acetic acid/0.2 M sodium acetate at 25 ${}^{\circ }$C and the corresponding viscosity average molecular weight, $M_{v}$, were listed in Table 3. The molecular weights of all samples were very similar in all cases, indicating that the various reaction conditions employed did not introduce substantial differences for the size of the chitosan chains.

### 3.5. X-ray Diffraction

**Figure 6:**
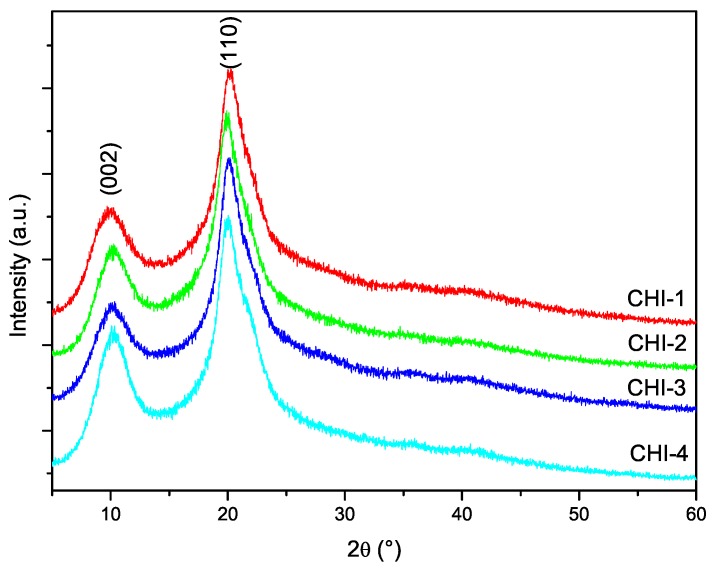
Powder X-ray diffraction patterns of chitosans prepared with the conditions listed in Table 1.

The diffractograms of the chitosan samples are shown in Figure 6. The characteristic peaks of chitosan centered at 10 and 20 degrees in 2θ corresponding to the crystallographic planes (002) and (101), respectively, are present in the diffractograms of all samples. The crystallinity index of chitosans was calculated from the diffractograms using Equation (6). It has been pointed out that this procedure might lead to an underestimation of CrI, since the contribution of the amorphous phase is overestimated. However, since the same procedure has been used for the all samples, the results can be used for comparison. The values listed in Table 3 indicate that CrI was around 40% for almost all chitosans.

## 4. Conclusions

In the procedure developed in the present work, chitosan was obtained as a white powder with adequate physical-chemical properties: ash content below 0.063%, and high solubility in 1% acetic acid, with insoluble contents of 0.62% or less. The deacetylation degrees achieved were above 90% and the crystallinity index were around 40%. The molecular weight of chitosans were between 2.3 and $2.8\times 10^{5}$ g/mol. The results obtained indicate that, with the present preparation procedure, chitosan samples with physical and chemical properties suitable for pharmaceutical applications are obtained.

## Figures and Tables

**Table 1:** Sulfated ash and insoluble content of chitosan samples prepared with the reaction times indicated. Deproteinization time was 3 h in all cases.

Sample	Demineralization Time (h)	Deacetylation Time (h)	Sulfated Ash (%) ± SD	Insoluble Content (%) ± SD
CHI-1	6	4	0.063±0.042	0.27±0.06
CHI-2	2	6	0.053±0.023	0.30±0.11
CHI-3	0.5	6	0.010±0.002	0.48±0.04
CHI-4	6	6	0.047±0.015	0.62±0.23

SD—Standard deviation.

**Table 2:** Degree of acetylation, DA (%), of chitosans obtained from FTIR spectroscopy (Equations (3)–(5)) and the UV-first derivative method.

Sample	FTIR			UV-First Derivate ± SD
	Equation (3) ± SD	Equation (4) ± SD	Equation (5) ± SD	
CHI-1	2.7±0.2	4.1±0.3	7.9±0.5	8.9±0.9
CHI-2	2.6±0.5	4.0±0.7	7.8±0.9	8.6±1.0
CHI-3	2.5±0.3	3.9±0.2	8.1±0.2	8.9±0.4
CHI-4	2.5±0.8	3.9±0.4	7.6±1.2	8.7±0.6

SD—Standard deviation.

**Table 3:** Intrinsic viscosity, [η], viscositiy average molecular weight, $M_{v}$ and crystallinity index, CrI (%) of chitosans.

Sample	[η] (100 mL/g) ± SD	$M_{v}$ (g/mol)	CrI (%)
CHI-1	1051±9	$2.8\times 10^{5}$	45
CHI-2	1030±19	$2.7\times 10^{5}$	40
CHI-3	898±19	$2.3\times 10^{5}$	40
CHI-4	998±6	$2.6\times 10^{5}$	46

SD—Standard deviation.

## Abbreviations

The following abbreviations are used in this manuscript:

MDPI	Multidisciplinary Digital Publishing Institute
DOAJ	Directory of Open Access Journals
DA	Acetylation Degree
EDS	Energy Dispersive Spectroscopy
ASTM	American Society for Testing and Materials
FTIR	Fourier Transform Infrared Spectroscopy
UV	UV-Visible Spectroscopy
XRD	X-Ray Diffraction
WAXD	Wide Angle X-Ray Diffraction
CrI	Crystallinity Index
SEM	Scanning Electron Microscope
SD	Standard Deviation
OPAS	Organization Pan American Health
CAPES	Coordination for the Improvement of Higher Education Personnel
